# Supplementary material for: Microbial Community Succession Associated with Poplar Wood Discoloration
Source: Plants (Basel). 2022 Sep 16;11(18):2420. doi: 10.3390/plants11182420 (PMC9504049; doi:10.3390/plants11182420)
Supplement: Supplementary file 1 [file plants-11-02420-s001.zip › plants-1928065-supplementary.pdf]

Microbial Community Succession Associated with Poplar Wood Discoloration

Xiaohua Zhang [1,2], Hao Liu [1], Heming Han [1], Bo Zhang [1], Cunzhi Zhang [1], Jian He [1], Shunpeng Li [1,*] and Hui Cao [1,*]

[1] Key Laboratory of Agricultural Environmental Microbiology, Ministry of Agriculture and Rural Affairs, College of Life Sciences, Nanjing Agricultural University, Nanjing 210095, China

[2] Bioengineering Technology Center, College of Tea and Food Science and Technology, Jiangsu Vocational College of Agriculture and Forestry, Nanjing 212499, China

* Correspondence: lsp@njau.edu.cn (S.L.); hcao@njau.edu.cn (H.C.)

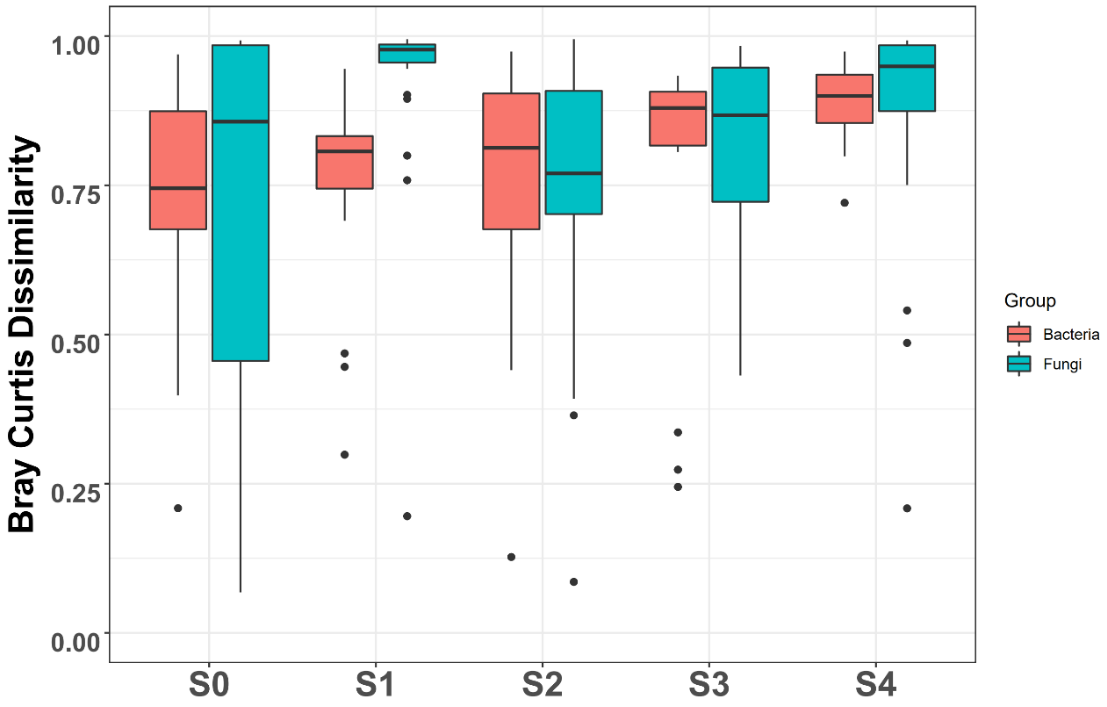

Figure S1. Bray Curtis dissimilarity of microbial communities among different discoloration stages of poplar wood.

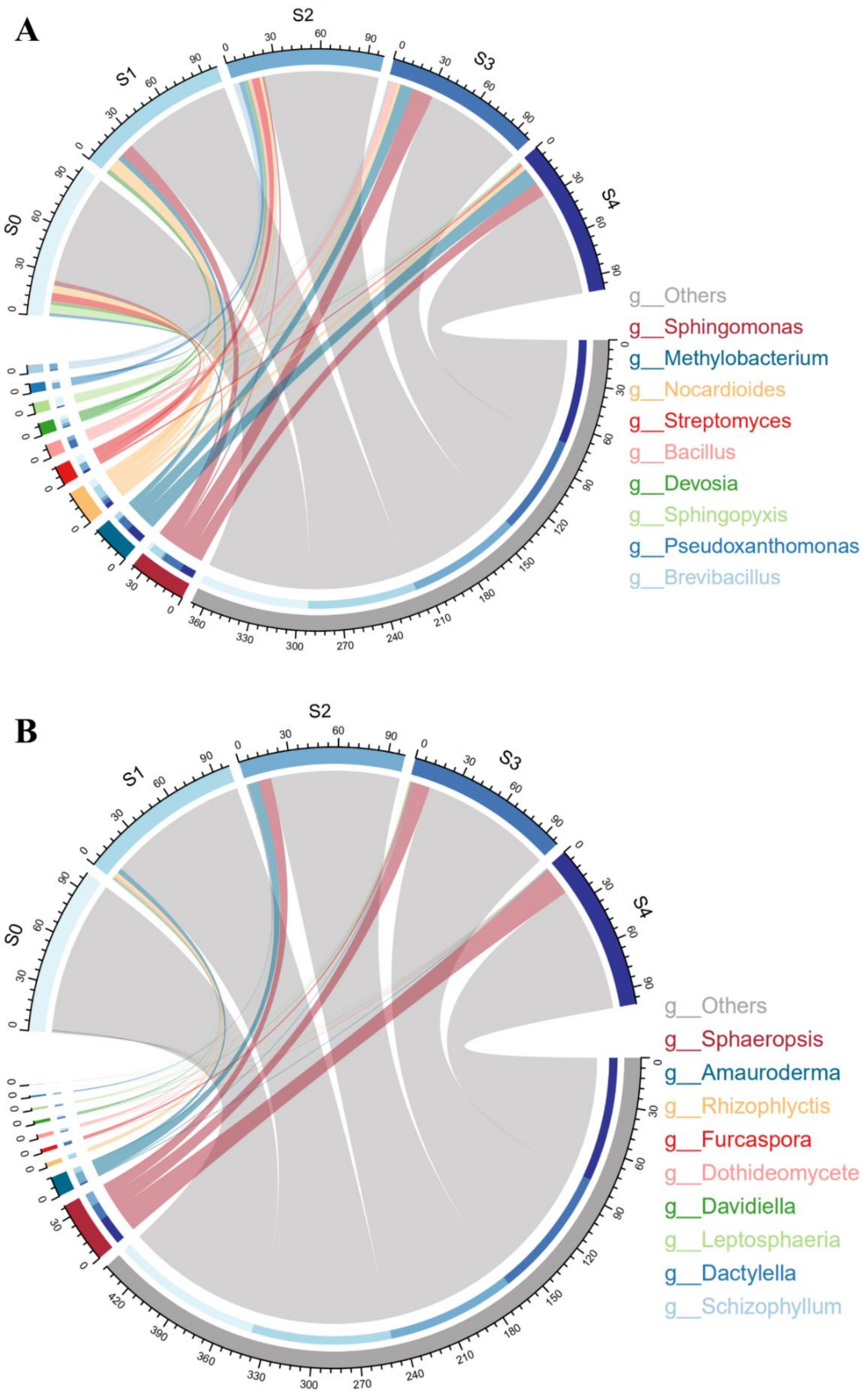

Figure S2. Composition of bacterial and fungal community on gene level of five groups. (A) composition of bacterial community; (B) composition of fungal community. The different colors represent the various taxa at gene level.
